# Depletion of mitochondrial protease OMA1 alters proliferative properties and promotes metastatic growth of breast cancer cells

**DOI:** 10.1038/s41598-019-49327-2

**Published:** 2019-10-14

**Authors:** Amita Daverey, Roman M. Levytskyy, Kimberly M. Stanke, Martonio Ponte Viana, Samantha Swenson, Stephen L. Hayward, Madhusudhanan Narasimhan, Oleh Khalimonchuk, Srivatsan Kidambi

**Affiliations:** 1 Department of Chemical and Biomolecular Engineering, University of Nebraska, Lincoln, NE United States; 2 Department of Biochemistry, University of Nebraska, Lincoln, NE United States; 3 Department of Pharmacology and Neuroscience, Texas Tech University Health Sciences Center, Lubbock, TX United States; 4 Nebraska Redox Biology Center, University of Nebraska, Lincoln, NE United States; 5 Fred & Pamela Buffett Cancer Center, University of Nebraska Medical Center, Omaha, NE United States; 6 Nebraska Center for Integrated Biomolecular Communication, University of Nebraska, Lincoln, NE United States; 7 Nebraska Center for the Prevention of Obesity Diseases, University of Nebraska, Lincoln, NE United States; 8 Nebraska Center for Materials and Nanoscience, University of Nebraska, Lincoln, NE United States; 9 Mary and Dick Holland Regenerative Medicine Program, University of Nebraska Medical Center, Omaha, NE United States

**Keywords:** Breast cancer, Cancer

## Abstract

Metastatic competence of cancer cells is influenced by many factors including metabolic alterations and changes in mitochondrial biogenesis and protein homeostasis. While it is generally accepted that mitochondria play important roles in tumorigenesis, the respective molecular events that regulate aberrant cancer cell proliferation remain to be clarified. Therefore, understanding the mechanisms underlying the role of mitochondria in cancer progression has potential implications in the development of new therapeutic strategies. We show that low expression of mitochondrial quality control protease OMA1 correlates with poor overall survival in breast cancer patients. Silencing OMA1 *in vitro* in patient-derived metastatic breast cancer cells isolated from the metastatic pleural effusion and atypical ductal hyperplasia mammary tumor specimens (21MT-1 and 21PT) enhances the formation of filopodia, increases cell proliferation (Ki67 expression), and induces epithelial-mesenchymal transition (EMT). Mechanistically, loss of OMA1 results in alterations in the mitochondrial protein homeostasis, as reflected by enhanced expression of canonic mitochondrial unfolded protein response genes. These changes significantly increase migratory properties in metastatic breast cancer cells, indicating that OMA1 plays a critical role in suppressing metastatic competence of breast tumors. Interestingly, these results were not observed in OMA1-depleted non-tumorigenic MCF10A mammary epithelial cells. This newly identified reduced activity/levels of OMA1 provides insights into the mechanisms leading to breast cancer development, promoting malignant progression of cancer cells and unfavorable clinical outcomes, which may represent possible prognostic markers and therapeutic targets for breast cancer treatment.

## Introduction

Breast cancer is a complex disease affecting over 180,000 women annually involving a continuously changing phenotype and microenvironment leading to differences in the gene and protein profile of the neoplasm^1–5^. Metastasis to vital organs including lungs, liver, bone, and brain is a major cause for breast cancer-related deaths^6^. Two main processes, migration and invasion, mediate the metastatic activity of tumor cells. Mounting evidence indicates that altered mitochondrial functions play a significant role(s) in the regulation of tumor cell biology and are likely involved in tumor progression including metastasis^7–12^. Growing evidence indicates that mitochondrial integrity is central to cancer cell physiology, particularly with regard to energy production and cell survival in the highly dynamic tumor environment. Perturbations to mitochondrial integrity by mutations or functional decline lead to mitochondrial dysfunction, potentially impacting physiological properties of neoplastic cells. Many cancer cells undergo the metabolic adaptation known as aerobic glycolysis, or Warburg effect; which involves enhanced utilization of glucose or pyruvate for anabolic processes underpinning rapid proliferation^12–14^. These cells also rely on glutamate anaplerosis to replenish the tricarboxylic acid (TCA) cycle with pyruvate. Alterations in mitochondrial function and the subsequent metabolic reprogramming are now being recognized as important hallmarks of malignancy and metastasis^12^. To date, several studies reported a loss of key mitochondrial proteins in various cancers, including breast cancer^15–17^. However, the mechanisms by which mitochondrial malfunction contributes to cancer progression remain far from clear.

Mitochondria are highly dynamic organelles whose form and shape are regulated through two critical processes: fission and fusion. The dynamic nature of mitochondrial networks allows the adjustment of mitochondrial morphology and metabolism to specific cellular processes and is also essential for mitochondrial protein quality control (MQC)^18–20^. MQC comprises a unique and conserved set of interrelated mechanisms critical for the organelle’s health^21^. Recent reports implicate several MQC modules in development and progression of various cancers^22^. For instance, elevated activities of ATP-driven proteases Lon proteases (LONP) and mitochondrial unfoldase-peptidase complex ClpXP have been shown to correlate with tumor development and progression^23,24^. Consequently, inhibition of these proteases has been proposed as a potential therapeutic strategy in patients with lymphoma and acute myeloid leukemia, respectively^22,25^. Activities of several other MQC proteases have been suggested to prevent malignant progression^26,27^; however, the molecular details of this process remain to be clarified.

Overlapping With The m-AAA Protease 1 (OMA1) is a conserved metallopeptidase that has recently emerged as a critical regulator of metabolic homeostasis^19,28–30^, mitophagy^31^, and apoptosis^32^. At least in part, such functional versatility is attributed to the enzyme’s ability to process the GTPase, Optic Atrophy Protein 1 (OPA1), and thus rapidly modulate the mitochondrial network^31^. Recently, we discovered that OMA1-deficient mouse embryonic fibroblasts exhibit increased aerobic glycolysis upon culturing conditions that require maximal bioenergetic output^33^. Intriguingly, results of the Human Protein Atlas Consortium initiative^15–17^ report low OMA1 protein levels in breast and testicular cancer tissues, as well as in lymphomas. However, whether OMA1 plays a role in the development and progression of breast adenocarcinoma is unknown.

In the present study, we report that depletion of OMA1 in stable, patient-derived breast cancer cells isolated from the metastatic pleural effusion (21MT-1) and atypical ductal hyperplasia (21PT) increased expression of the canonical mitochondrial unfolded protein response (UPR^mt^) markers, cell spreading, and filopodia formation. Moreover, sustained silencing of OMA1 resulted in a large proportion of cells exhibiting mesenchyme-like morphology, reduced proliferation, and enhanced migratory properties indicative of the epithelial-mesenchyme transition (EMT). Consistent with enhanced metastatic abilities of the OMA1-depleted breast cancer cells, we observed a significant upregulation of mesenchymal markers and decreased expression of epithelial markers at both the gene and protein levels. Together with bioinformatics data indicating that breast cancer patients with low, EMT markers-correlating OMA1 expression have a shorter survival time, our findings led us to conclude that impaired MQC function through OMA1 deficit can regulate malignancy and metastatic progression in breast cancer.

## Results

### OMA1 expression levels correlate with patient survival in breast cancer

**Figure 1:**
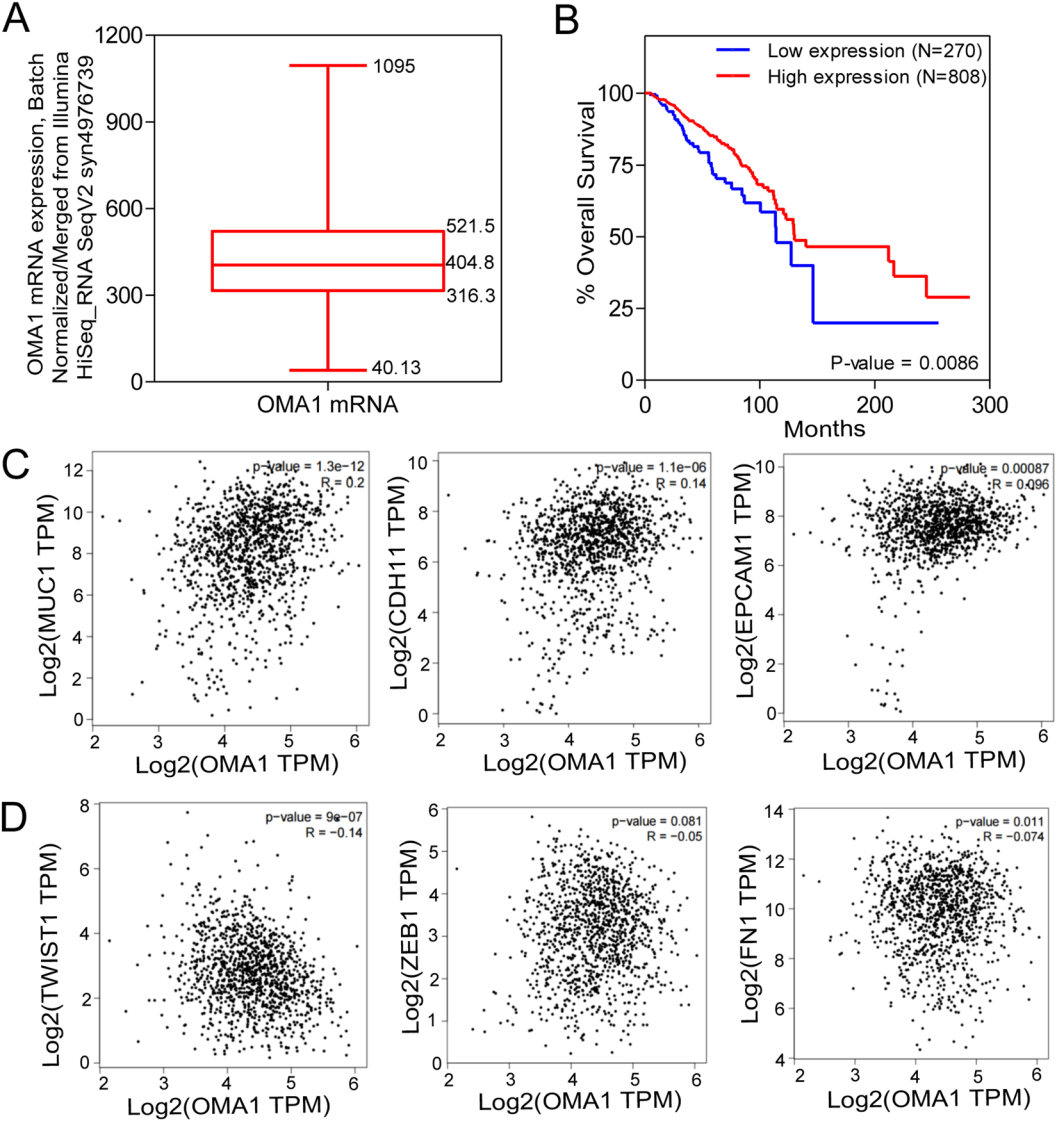
OMA1 expression levels correlate with patient survival in breast cancer. (**A**) OMA1 mRNA expression is represented as batch normalized/merged from Illumina HiSeq_RNASeqV2 syn4976369 generated by The Cancer Genome Atlas (TCGA). Out of 1084, the expression data was available for 1082 samples. Normal distribution across the dataset is visualized with box and whisker plots, shown as 25^th^ (316.3) and 75^th^ (521.5) percentiles with a median of 404.8. From this a 316.3 expression cut-off was assigned to evaluate the Survival. (**B**) Out of 1084 samples, only 1078 patients had all the information required for Survival analysis. Among these 1078 patients that had all the information, based on the OMA1 expression (at or below 25^th^ percentile assigned as low and above 25^th^ percentile assigned as high) was used to compute the survival analysis. It was found that the patients with low OMA1 expression had poor survival when compared to patients with high OMA1-expression. The number of events (deaths) were ~17% (46/270) in the group with low OMA1 expression, while the number of events in the group with high OMA1 expression is only ~13% (105/808), which comprises a ~31% increase in the mortality in the group expressing low levels of OMA1 in comparison to the group expressing high levels of OMA1. (**C**) Pearson’s correlation analysis of the mRNA expression datasets for OMA1 and epithelial marker genes-MUC1, CDH1, EPCAM using the input, TCGA_Tumor (BRCA Tumor) and TCGA_Normal (BRCA_Normal). (**D**) Pearson’s correlation analysis of the mRNA expression datasets for OMA1 and mesenchymal marker genes-TWIST1, ZEB1, Fibronectin (FN1) using the input, TCGA_Tumor (BRCA Tumor) and TCGA_Normal (BRCA_Normal).

To determine if OMA1 expression was related to patient prognosis, we performed survival risk prediction using survival database to evaluate the correlation between OMA1 expression levels and overall survival rates in breast cancer patients (Fig. 1). OMA1 mRNA expression is represented as batch normalized/merged from Illumina HiSeq_RNASeqV2 syn4976369 generated by “The Cancer Genome Atlas (TCGA)”. Out of 1084 breast cancer patient samples, the expression data were available for 1082 samples. Normal distribution across the dataset is visualized with box and whisker plots, shown as 25^th^ (316.3) and 75^th^ (521.5) percentiles with a median of 404.8 (Fig. 1A). Based on this data and reports from Geng *et al*.^31^ and Bertucci *et al*.^32^, we dichotomized the samples into low OMA1 and high OMA1 expressing group with values up to 25^th^ percentile (≤316.3) comprising the former and those equaling and above the 25^th^ percentile (316.3) into the latter.

To better understand the involvement of OMA1 in breast cancer, we next determined the survival of subjects in the low and high OMA1 expressing subsets. Out of 1084 samples, 1078 had all the necessary details such as OMA1 gene expression, overall survival months, and vital status to perform Kaplan-Meier survival analysis (Fig. 1B). In these cohort of 1078 samples that had all the available survival data and applying the above dichotomy rule, 270 samples stratified into low and 808 samples segregated into high OMA1 expressing subset. Survival plot was generated using the OMA1-segregated samples. It was found that the OMA1-low expressing patients had poor survival when compared to OMA1-high expressing patients. The number of events (deaths) was ~17% (46/270) in the low-OMA1 expressing group, while the number of events in the high-OMA1 expressing group is only ~13% (105/808), indicating that low OMA1 expression increased the probability of mortality by 31% when compared to the high-OMA1 expressing subset. In parallel, the median survival was 114.06 and 130.06 months in OMA1-low and –high expressing patients, respectively. In other words, the OMA1-low expressing patients displayed a ~14% decrease in the median survival when compared to OMA1-high expressing subjects. These results suggest that a low OMA1 expression could be associated with poor overall survival in breast cancer.

Typically, epithelial to mesenchymal transition (EMT) program is a process where cells lose epithelial traits (decreased marker expression) and acquire mesenchymal characteristics (increased marker expression) that is tightly associated with malignancy, invasion, metastasis, and poor outcome^34–36^. While this generally applies to many transformed cells, we posited that attenuation of OMA1 might facilitate EMT program. To that end, we used GEPIA (Gene Expression Profiling Interactive Analysis), a web-based tool (uses TCGA and GTEx dataset) to determine the relationship between aberrant OMA1 mRNA levels with the expression of some of the common EMT marker genes in human breast cancer (Fig. 1C,D). Pearson correlation analysis of the mRNA expression datasets for OMA1 and epithelial marker genes such as MUC1, CDH1, and EPCAM using the input, TCGA_Tumor (BRCA Tumor) and TCGA_Normal (BRCA_Normal) revealed a statistically significant positive correlation with the OMA1 mRNA expression (P = 1.3e^−12^, R = 0.2 for MUC1; P = 1.1e^−06^, R = 0.14 for E-cadherin (CDH1); P = 0.00087, R = 0.096 for EPCAM) in the TCGA_BRCA dataset when compared to the TCGA_Normal dataset (Fig. 1C). This implies that OMA1 presence could promote epithelial signals and trait. In other words, depletion of OMA1 can be correlated to a loss of epithelial morphology. Similar correlation analysis was conducted for mRNA expression datasets for OMA1 and mesenchymal marker genes such as TWIST1, ZEB1, and Fibronectin (FN1) using the input, TCGA_Tumor (BRCA Tumor) and TCGA_Normal (BRCA_Normal) with Pearson’s statistical test (Fig. 1D). In contrast, to the changes observed in epithelial markers, all the analyzed mesenchymal markers are negatively correlated with OMA1 mRNA expression as can be seen from the negative R values (−0.14, −0.05, −0.074 for TWIST1, ZEB1, and Fibronectin (FN1), respectively) from the TCGA_BRCA vs TCGA_Normal dataset comparison. Moreover, OMA1 expression displayed significant difference with the TWIST1 (P = 9.0e^−07^) and FN1 expression (P = 0.11) with ZEB1 tending towards significance (P = 0.081). This denotes that the presence of OMA1 could repress mesenchymal signals and phenotype. In other words, the decreased OMA1 expression can be correlated to a gain in mesenchymal characteristics. These clinical data demonstrates that attenuated OMA1 levels in human breast cancer patients are correlated to the molecular gene signatures buttressing EMT, a well-established hallmark of invasion/metastasis. Importantly, to the best of our knowledge, this is the first study that has evaluated the clinical value of OMA1 in breast cancer, particularly in the context of EMT process.

### OMA1-deficient cells exhibit signs of metabolic reprogramming

**Figure 2:**
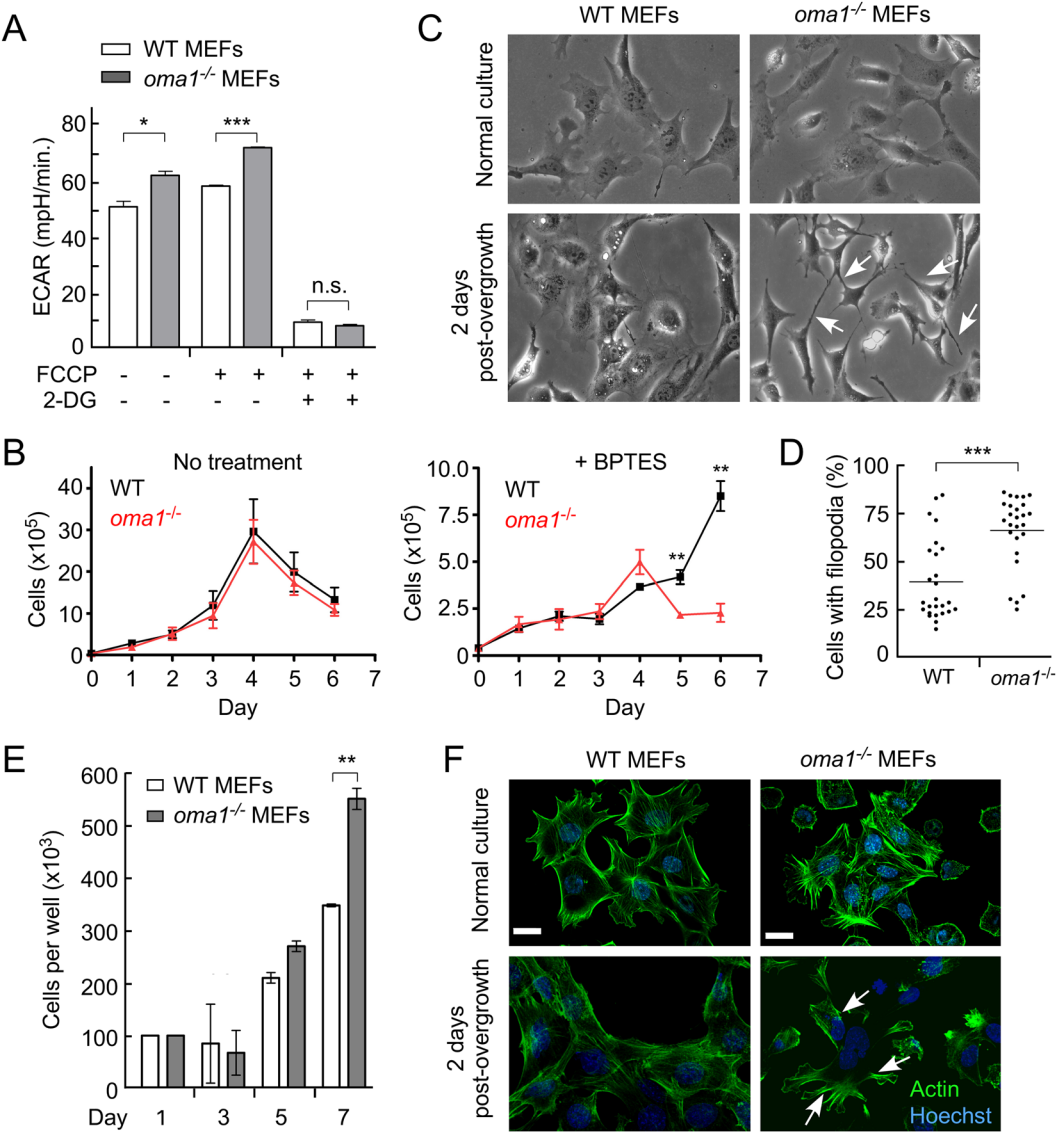
Loss of OMA1 alters metabolic and proliferative properties of mouse embryonic fibroblasts. (**A**) Extracellular acidification rates (ECAR) in wild type and *oma1*^−/−^ mouse embryonic fibroblasts under basal, FCCP-stimulated, and glycolysis-ablating (2-DG) conditions. Cells were cultured in the media containing 10 mM glucose. Data are shown as mean values of 3 independent experiments ± S.E.M.; **p < 0.01, by unpaired *t*-test. (**B**) Growth rate of wild type (WT) and *oma1*^−/−^ MEF cells under conditions of glutamate pathway inhibition by BPTES. Cells were cultured in the 10 mM galactose-containing medium with (left panel) or without (right panel) 10 μM BPTES for the indicated periods of time and the number of viable cells at each time point has been assessed. Data represent the mean ± S.D. (n = 3 biological replicates); **p < 0.01, by unpaired *t*-test. (**C**) Live phase contrast images of MEF WT and *oma1*^−/−^ cells at 2 days after seeding in normal culture conditions and after overgrowth for 7 days at 100% confluence. Unlike the WT cells, post-quiescent *oma1*^−/−^ MEFs exhibit characteristic filopodia-like protrusions (white arrows) after overgrowth. (**D**) The prevalence of cells with protrusions was analyzed in WT and *oma1*^−/−^ MEF cells two days after re-seeding on 6-well plates after one week of growth at 100% confluence. Each well was randomly imaged in 2–3 fields of view, each containing 15–50 cells. In the randomly chosen imaging fields, the number of cells with long filopodia and cells with normal filopodia were counted. The data was plotted as a scatter plot where each point represents percentage of cells with long filopodia of total cells in one field of view. (**E**) Proliferation of MEF WT and *oma1*^−/−^ cells seeded after overgrowth of 7 days at 100% confluence. Data represent the mean ± S.D. of n = 3 biological replicates; **p < 0.01. (**F**) Paraformaldehyde-fixed WT and *oma1*^−/−^ MEF cells on 2nd day after seeding post-overgrowth of 7 days at 100% confluence. Samples were stained for Actin with Alexa Fluor 488-conjugated phalloidin and imaged by confocal microscopy. The arrows mark rearranged actin patches. Scale bar, 20 μm.

Our recent study revealed that upon culturing in low glucose medium, OMA1-deficient mouse embryonic fibroblasts (MEFs) are compromised in their ability to maximize their bioenergetic output and appear to rely on compensatory aerobic glycolysis, as indicated by an increased extracellular acidification rate ([28] and Fig. 2A). This observation likely reflects partial metabolic reprogramming and suggests that *oma1*^−/−^ MEFs are probably more reliant on anaplerotic metabolism. As such, these cells are expected to replenish their citrate levels through enhanced utilization of glutamine that is metabolized via reductive carboxylation – enzymatic conversion of glutamine to glutamate, which is then modified to α-ketoglutarate, followed by its conversion to oxaloacetate and then citrate [29, 30]. Thus, OMA1-deficient cells are expected to be more sensitive to inhibition of glutaminase (GLS) – a key enzyme mediating the first step in the above chain of reactions. To test this postulate, we compared the proliferation of wild type and *oma1*^−/−^ cells cultured in either low glucose or galactose-containing medium in the presence or absence of 10 μM bis-2-(5- phenylacetamido-1,2,4-thiadiazol-2-yl)ethyl sulfide (BPTES) – a specific small molecule inhibitor of GLS. While the proliferation of the cells in question was nearly identical under normal conditions, *oma1*^−/−^ MEFs proliferated at much slower rates than wild type cells in the presence of BPTES (Fig. 2B). These observations prompted us to examine the ability of the WT and *oma1*^−/−^ MEFs to re-enter the proliferative cycle after aging-induced quiescence, a process known to require normal mitochondrial function [31]. Contact-inhibited quiescent WT and *oma1*^−/−^ MEFs were cultured for 7 days with fresh medium added every 2 days. Following such incubation, the proliferative properties of these post-quiescent cells were tested. While growth characteristics and morphology of the young WT and *oma1*^−/−^ cells were virtually indistinguishable (Fig. 2C), the post-quiescent *oma1*^−/−^ MEFs formed filapodia -like structures (Fig. 2C,D) and proliferated significantly faster than control MEFs (Fig. 2E). Likewise, the post-quiescent growth of *oma1*^−/−^ MEFs was characterized by enhanced cell spreading when compared to the wild type control. Moreover, consistent with the observed filapodia structures, post-quiescent *oma1*^−/−^ MEFs displayed abnormal actin distribution patterns (Fig. 2F) resembling rearrangements reported in neoplasms [32]. These results further highlight metabolic and morphological alterations associated with proliferative activity in OMA1-deficient mammalian cells.

### Loss of OMA1 causes filopodia formation and mitochondrial fragmentation in human breast adenocarcinoma cell lines

**Figure 3:**
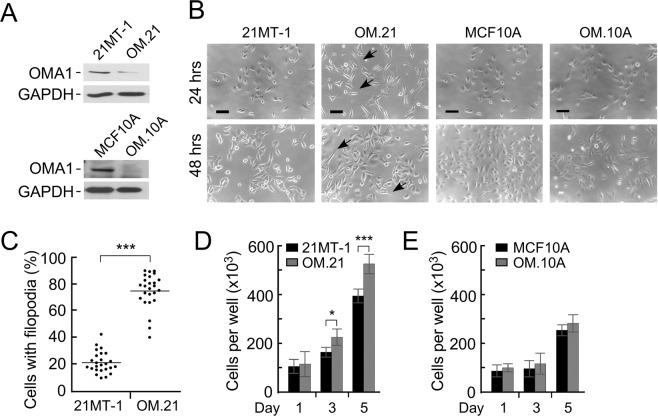
OMA1 depletion in metastatic breast cancer cells promotes filopodia formation. (**A**) The *top panel* shows a representative Western blot of extracts from the metastatic pleural effusion mammary tumor 21MT-1 cells and MCF10A cells before and after transfection with either scrambled shRNA (control) or shRNA directed against OMA1 (OM.21 and OM.10 A). Steady-state levels of the protease were detected by immunoblotting with anti-OMA1. The GAPDH blots served as a loading control. (**B**) Live phase contrast images of 21MT-1 cells and MCF10A cells before and after transfection with scrambled shRNA (control) or shRNA directed against OMA1 (OM.21 and OM.10A) at 24 h and 48 h after seeding in normal culture conditions. Unlike the control cells, OM.21 cells exhibit characteristic filopodia-like structures (black arrows). The arrows indicate filopodia. Scale bar, 20 μm. (**C**) The prevalence of cells with protrusions was analyzed in 21MT-1 cells and OM.21 cells two days after re-seeding on 6-well plates after one week of growth at 100% confluence. Each well was randomly imaged in 3–4 fields of view, each containing 40–75 cells. In the randomly chosen fields of view, the number of cells with long filopodia and cells without these structures were counted. The data was plotted as a scatter plot where each point represents percentage of cells with long filopodia of total cells in one field of view. (**D**) Proliferation of 21MT-1 WT and OM.21 cells seeded after overgrowth of 7 days at 100% confluence. Data represent the mean ± S.D. of n = 3 biological replicates; *p < 0.05, ***p < 0.001. (**E**) Proliferation of MCF10A WT and OMA.10 A cells seeded after overgrowth of 7 days at 100% confluence. Data represent the mean ± S.D. of n = 3 biological replicates.

Alteration of focal adhesions is a hallmark of tumorigenesis [32]. The results of our analyses in MEFs suggest that loss of OMA1 may be related to neoplastic development and/or progression. Moreover, OMA1 protein levels were reported to be extremely low in breast and testicular cancer tissues, as well as lymphomas^15–17^. We thus hypothesized that OMA1 function may be attenuated in these cancers and such a setting could be an important contributor to malignancy. To test this hypothesis, we generated stable knockdowns of OMA1 in non-tumorigenic breast epithelial cells (MCF10A) and patient-derived breast cancer cells isolated from metastatic pleural effusion mammary tumor specimen (21MT-1) cells using OMA1-specifc shRNAs. Immunoblotting with an anti-OMA1 antibody confirmed efficient depletion of the protease in cells expressing OMA1 shRNA but not the scrambled control (Fig. 3A and Supplementary Fig. S1). Transfection of 21MT-1 cells with OMA1-targeting shRNAs caused over 80% reduction in endogenous OMA1 protein as compared to control cells transfected with scrambled shRNAs. Microscopic images of OMA1-depleted 21MT-1 (hereafter referred to as OM.21) cells revealed enhanced filopodia formation as early as 24 hours of culture that was even more pronounced after 48 hours (Fig. 3B,C and Supplementary Fig. S2). Remarkably, this was not the case for OMA1-depleted MCF10A cells (designated OM.10 A; Fig. 3B). Also, OM.21 cells demonstrated higher proliferation rates compared to control 21MT-1 cells while no significant change in proliferation rate was observed in OM.10A and control MCF10A cells (Fig. 3D,E). Strikingly, these findings parallel our observations in *oma1*^−/−^ MEFs. To further extend these findings in other cancer cells, we generated stable knockdowns of OMA1 in patient-derived breast cancer cells isolated from atypical ductal hyperplasia (21PT) using OMA1-specific shRNAs. Immunoblotting with an anti-OMA1 antibody confirmed a robust depletion of the endogenous levels of this protease by about 80% in cells expressing OMA1 shRNA when compared to control (Supplementary Fig. S3A). Like 21MT-1, an enhanced filopodia formation was observed as early as 24 hours of culturing the OMA1-depleted 21PT (hereafter OP.21) cells, while this effect was more pronounced after 48 hours of culturing (Supplementary Fig. S3B–D). These observations replicate our findings in the 21MT-1 cells.

**Figure 4:**
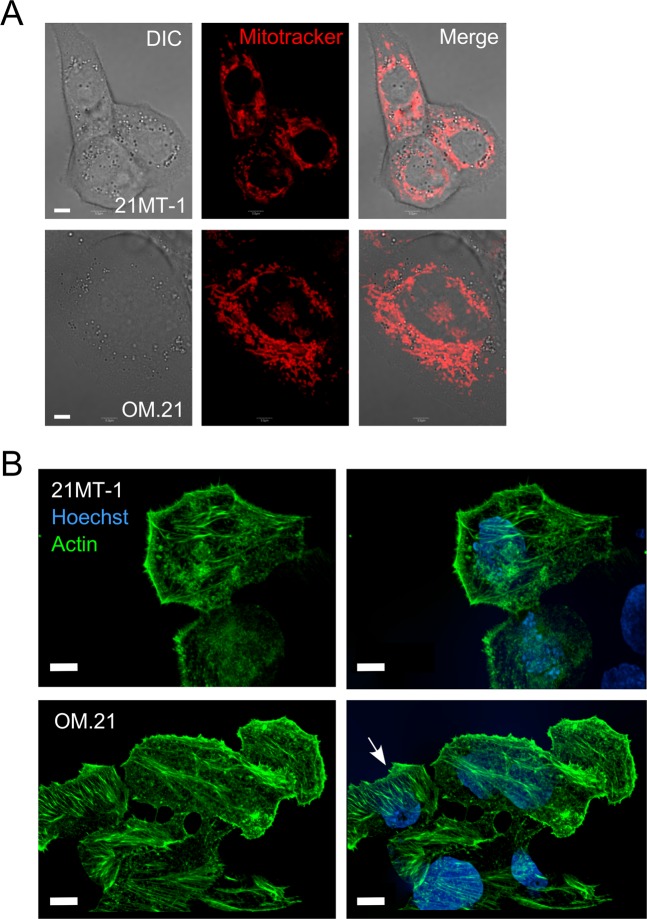
Loss of OMA1 alters mitochondrial network organization and actin branching in 21MT-1 cells. (**A**) Visualization of mitochondria in 21MT-1 and OM.21 cells. Image of MitoTrackerR Red CMXRos-stained mitochondria in each respective cell type were captured using confocal fluorescent microscopy, Scale bar, 5 μm. (**B**) Confocal fluorescent images of immunostained actin fibers in paraformaldehyde-fixed control and OM.21 cells show actin branching in the OMA1-depleted breast cancer cells, Scale bar, 5 μm.

To probe and visualize the effect of OMA1 knockdown on mitochondrial morphology and integrity, we imaged the mitochondria of OMA1-silenced 21MT-1 cells using the fluorescent stain Mitotracker FM (Fig. 4A). A typical fiber-like morphology was observed in control 21MT-1 cells indicating healthy mitochondria. In contrast, a slightly more dense or bulky mitochondrial network with perinuclear clustering was observed in OM.21 cells (Fig. 4A). In addition to differences in the spatial distribution and clustering of the mitochondrial reticulum seen in the OM.21 cells, the mitochondria within the clusters appeared to be slightly thicker and shorter segments in comparison to control. Such changes in mitochondrial network morphology likely reflect alterations in the mitochondrial dynamics and/or cristae remodeling associated with inactivation of OMA1-mediated processing of the IM GTPase, OPA1. Of note, and in line with our observation in *oma1*^−/−^ MEFs, the steady-state levels of the key components of mitochondrial fusion (mitofusins MFN1 and MFN2, and long and short isoforms of OPA1) and fission (DRP1) machinery remained largely unaltered in OM.21 (data not shown). We further investigated the impact of OMA1 knockdown on 21MT-1 cell morphology by immunostaining of the actin cytoskeletal structures (Fig. 4B). Remodeling of actin cytoskeleton influences cell migration and invasion^37^. Genetic depletion of OMA1 led to a substantial change in the 21MT-1 cell appearance, resulting in larger cell bodies and a stretched morphology. Additionally, the OM.21 cells exhibited considerably more stress actin fibers traversing the cytoplasm than in the control 21MT-1 cells. Interestingly, these effects were not observed in OM.10 A cells. Akin to *oma1*^−/−^ MEFs [28], the bioenergetics profiling of OM.21 cells revealed these cells were unable to maximize their respiration in response to mild uncoupling (Supplementary Fig. S4) and had significantly lower respiratory control ratios when cultured in the respiration-enforcing medium containing 10 mM galactose. Moreover, just like *oma1*^−/−^ MEFs, OM.21 cells exhibited a marked increase in sensitivity to the glutaminase inhibitor BPTES; all indicative of a bioenergetic deficit. These data suggest that loss of OMA1 promotes filopodia formation and significant remodeling of the cell morphology and mitochondrial physiology in breast cancer cells.

### Suppression of OMA1 increases proliferation and migration of metastatic breast adenocarcinoma cells

**Figure 5:**
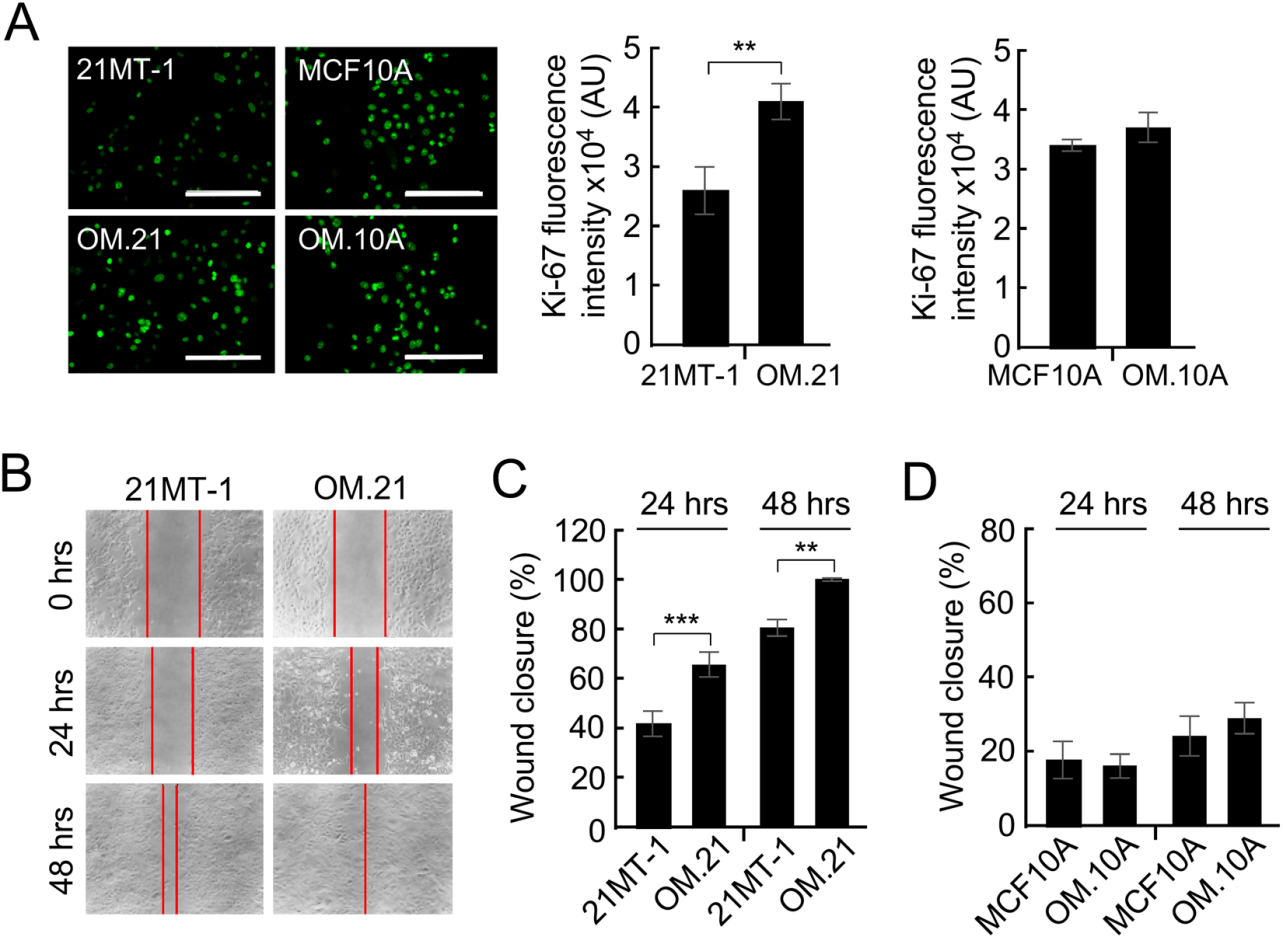
OMA1 enhances proliferation and migration of 21MT-1 metastatic breast adenocarcinoma cells. (**A**) Fluorescent images show Ki67 staining in MCF10A, OM.10A, 21MT-1, and OM.21 cells. Scale bar, 200 μm. Histogram shows increase in Ki67 expression in OM.21 cells compared to control cells after 2 days in culture. There is no significant difference in Ki67 expression in OM.10A and control MCF10A cells. At least three independent experiments were conducted for each analysis. (**B**) Data illustrating the migration of cells out from a confluent monolayer onto a featureless scratch or wound. Representative phase images of 21MT-1and OM.21 cells at 24 h and 48 h are shown. Scale bar, 20 μm. (**C**) Quantitative analysis of migratory properties of 21MT-1 and OM.21 cells. The extent of wound closure has been calculated as follows: % Wound Closure = [(Area at 0 h – Area at 24 or 48 h)/Area at 0 h] x 100%. Bars show mean data ± S.D. of 3 biological replicates; **p < 0.01, ***p < 0.001 by unpaired *t*-test. (**D**) Migratory properties of MCF10A and OM.10A cells were analyzed as in Fig. 4C.

The proliferation and filopodia-like structures play a key role in cancer cell invasion^38,39^. Increased appearance of these structures in OM.21 cells may indicate enhanced invasiveness of these cells and deregulation of the cell cycle. We examined this hypothesis by immunostaining 21MT1, MCF10A, OM.21, and OM.10A cells with Ki67 antibodies (Fig. 5A). Ki67, a nuclear protein, is a commonly used clinical marker to determine the proliferative state of tumor cells^3,4,40,41^. Remarkably, the Ki67 expression was higher in the OM.21 cells, indicating that these cells are in a hyperproliferative state relative to normal 21MT-1 cells (Fig. 5A). Interestingly, OM.10 A cells did not have any significant effect in the Ki67 expression as compared to control cells, indicating that this phenomenon is specific to neoplasms.

To further interrogate the intriguing link between OMA1 silencing and the observed enhancement of cellular migration, we examined the migratory potential of the metastatic breast cancer cells with normal and reduced levels of the protease. To this end, a scratch was made in a sub-confluent cell monolayer in control and OM.21 cells and cells were allowed to migrate into the cell-free area. Loss of OMA1 resulted in significantly higher migration in 21MT-1 cells at both 24 and 48 hours compared to controls (Fig. 5B,C). In contrast, OMA1 depletion had no effect on the migratory properties of non-cancerous MCF10A cells (Fig. 5D). Similar to the results observed in 21MT-1 cells, abrogation of OMA1 resulted in significantly higher migration in 21PT cells at both 24 and 48 hours compared to controls (Supplementary Fig. S3E,F). These data suggest that loss of OMA1 function in pre-metastatic breast adenocarcinoma cells could potentially facilitate the metastatic behavior of the tumor likely via promoting the migration of breast cancer cells.

### OMA1 deficiency augments invasiveness of breast cancer cells by inducing epithelial to mesenchymal transition

**Figure 6:**
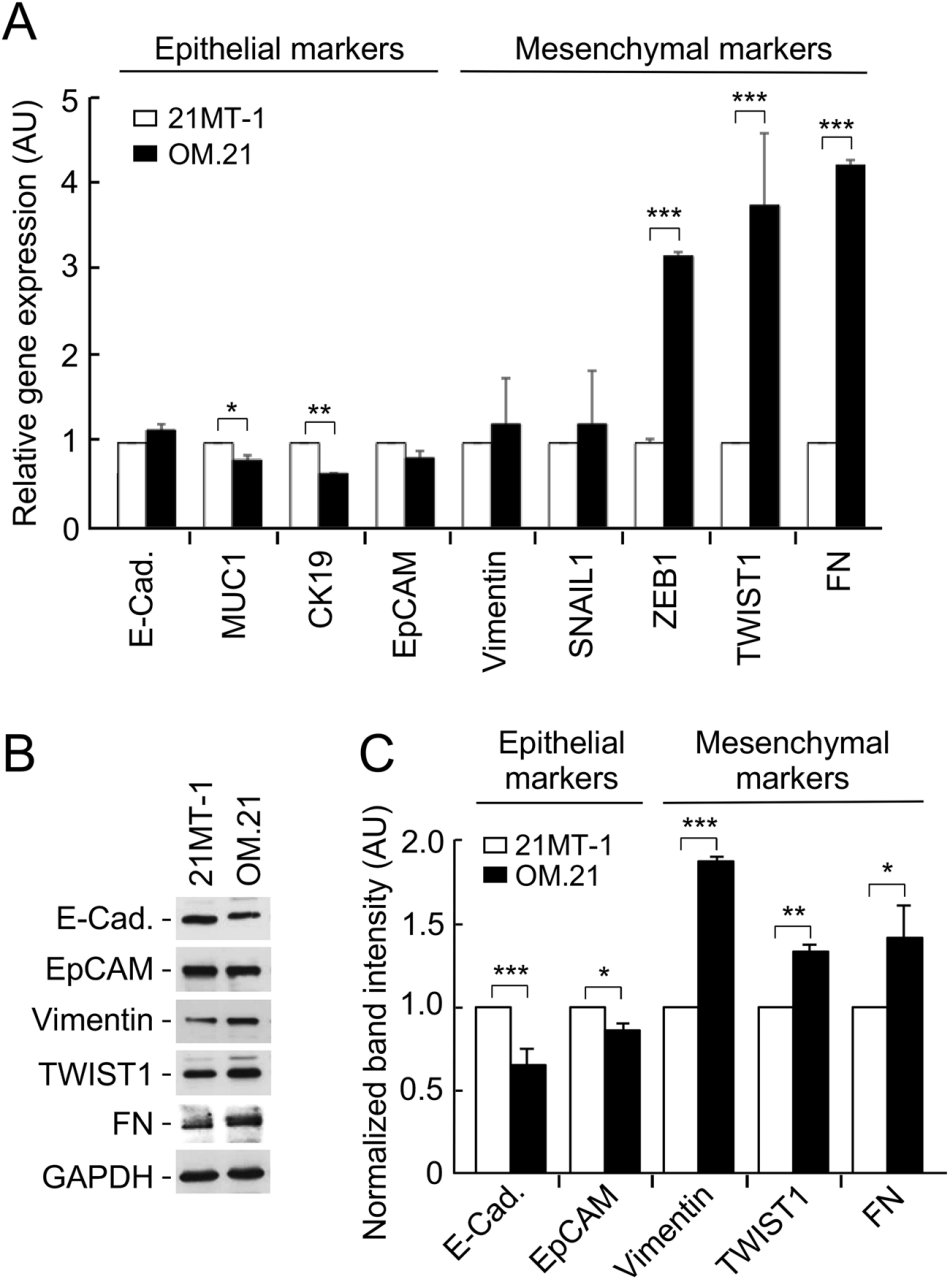
OMA1 deficiency enhances invasiveness of breast cancer cells by inducing epithelial to mesenchymal transition (EMT). (**A**) qRT-PCR analysis of the expression of EMT marker genes in 21MT-1 and OM.21 cells after 2 days in culture. The expression levels were normalized to GAPDH and to control cells. Data is presented as mean ± S.D. from 3 biological replicates. *p < 0.05; **p < 0.01; ***p < 0.001 by Student’s *t*-test. (**B**) Immunoblots showing protein expression of indicated EMT markers in 21MT-1 and OM.21 cells. (**C**) Densitometry analysis of the respective bands normalized after loading control (GAPDH) correction. Control cells grown served as control. Data are mean ± S.D.; n = 3 independent experiments; *p < 0.05; **p < 0.01; ***p < 0.001 by Student’s *t*-test.

We next investigated whether depletion of OMA1 promotes EMT in tumor cells. EMT is a transient process during which epithelial cancer cells acquire molecular alterations that facilitate the loss of epithelial features and gain a mesenchymal phenotype. This process is implicated during developmental stages and carcinogenesis and is characterized by phenotypic and molecular changes leading to increased invasive and metastatic capabilities of cancer cells and drug resistance. When compared to control, OM.21 cells had a significantly lower gene expression of epithelial markers, cytokeratin 19 (CK19) (1.5-fold) and MUC1 (2-fold), indicating a loss in epithelial features of the cell (Fig. 6A). Reciprocally, OM.21 cells displayed substantially higher expression levels of key mesenchymal markers: ZEB1 (3-fold), TWIST1 (3.5-fold), and fibronectin (FN) (4-fold) (Fig. 6A). We further examined the protein levels of some of the EMT markers by immunoblotting (Fig. 6B,C). A similar trend was observed with a significant decrease in epithelial markers (E-cadherin, EpCAM) and an increase in mesenchymal markers (Vimentin, FN, and TWIST1) in the OM.21 cells (Fig. 6B,C). We also observed similar trends in 21PT cells where the loss of OMA1 resulted in significantly lower gene expression of epithelial markers, E-cadherin (4-fold) and MUC1 (1.5-fold), indicating a loss in epithelial features of the cell (Supplementary Fig. S3G). Reciprocally, OP.21 cells displayed substantially higher expression levels of key mesenchymal markers: Vimentin (3-fold), SNAIL1 (3.5-fold), and fibronectin (FN) (2-fold) (Supplementary Fig. S3G). Overall, these results suggest a plausible role of OMA1 in suppressing aggressiveness and metastatic potential of these breast cancer cells.

### Loss of OMA1 induces expression of canonical UPR^mt^ genes in breast cancer cells

To gain insight into the potential mechanisms underpinning enhanced proliferative capacities of metastatic OM.21 cells, we tested several potential scenarios. We ruled out the involvement of reactive oxygen species (ROS) and hypoxic signaling as OM.21 cells exhibited neither elevated ROS production, nor appreciable stabilization of HIF1α, relative to control cell lines (data not shown). Similarly, no significant changes in the nuclear to mitochondrial DNA ratio were observed in these cells – likely reflective of no alterations in mitochondrial proliferation and content.

**Figure 7:**
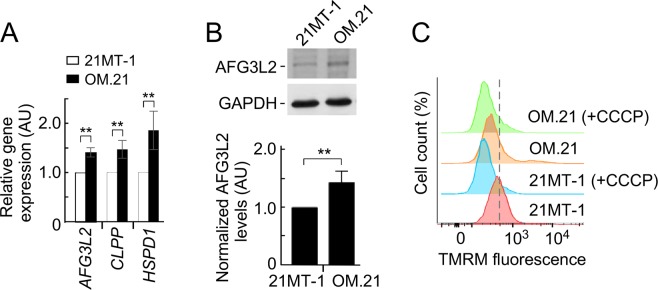
Loss of OMA1 in 21MT-1 cells regulates mitochondrial unfolded protein response. (**A**) qRT-PCR analysis showing increased expression of canonical UPR^mt^ genes *AFG3L2*, *CLPP*, and *HSPD1* in 21MT-1 and OM.21 cells. n = 3 independent experiments. Error bars show mean values ± S.E.M.; **p < 0.01, by Student’s *t*-test. (**B**) Representative immunoblots of extracts from 21MT-1 and OM.21 cells. Steady-state levels of the AFG3L2 were detected with the respective antibodies. The GAPDH served as a loading control. The bottom graphs show quantitative assessment of the immunoblots. Error bars, mean ± S.E.M.; *n*.*s*., **p < 0.01, by Student’s *t*-test. (**C**) Representative flow cytometry histograms of 21MT-1 and OM.21 cells incubated with 50 nM TMRM under basal and CCCP-induced uncoupling conditions. 50,000 cells were assayed for TMRM fluorescence in each experiment.

Metabolic adaptation to promote survival or proliferation of cells with mitochondrial dysfunction could be related to the mitochondrial unfolded protein response (UPR^mt^) [41]. We, therefore, tested if the loss of OMA1 results in UPR^mt^ activation. Quantitative real-time PCR and immunoblot analyses revealed that the transcript (Fig. 7A) and protein (Fig. 7B) levels of several canonical UPR^mt^ markers were significantly increased in OMA1-depleted 21MT-1 cells, respectively. The enhanced UPR^mt^ is consistent with a substantial decrease in the basal mitochondrial membrane potential in OM.21 cells (Fig. 7C), which is likely associated with attenuation of mitochondrial protein import and enhanced stabilization of UPR^mt^-mediating transcription factors such as ATF5 [42]. Akin to OM.21, we observed decrease in mitochondrial membrane potential in *oma1*^−/−^ MEFs as well (Supplementary Fig. S6). These data suggest that loss of OMA1 leads to UPR^mt^ activation and that sustained up-regulation of this signaling pathway is a likely contributor to the enhanced invasiveness of metastatic breast cancer cells.

## Discussion

In the present study, we have identified a novel role of the mitochondrial metallopeptidase OMA1 in the regulation of tumor progression in breast cancer cells. Our results show that depletion of OMA1 enhances the formation of filopodia, increased Ki67 expression, and induction of EMT markers. Concurrently, loss of OMA1 is associated with alterations in mitochondrial protein homeostasis, as reflected by enhanced expression of canonic mitochondrial unfolded protein response genes. These changes significantly increase migratory properties in metastatic breast cancer cells, indicating that OMA1 plays a critical mechanistic role in suppressing metastatic competence of breast tumors.

OMA1 is a conserved, stress-activated protease that resides in the IM and has been implicated in a number of mitochondrial processes - most notably stress-triggered regulation of mitochondrial dynamics^19,31^. Recent reports link OMA1 activity to the regulation of bioenergetic and metabolic outputs^28,30,33^, and demonstrated that loss of OMA1 in mouse embryonic fibroblasts leads to enhanced compensatory glycolysis^33^. In line with these observations, OMA1-depleted 21MT-1 cells exhibit hallmarks of metabolic remodeling, as indicated by greatly increased sensitivity of these cells to the glutaminase inhibitor, BPTES. Metabolic remodeling and formation of filopodia are hallmarks of many invasive epithelial tumors^14,38,42^. Our subsequent analysis of the OMA1 stable knockdown 21MT-1 primary metastatic breast ductal carcinoma and 21 PT atypical ductal hyperplasia cells recapitulated these observations, indicating a functional link between OMA1 and mechanisms that prevent EMT transition, and related metastatic properties.

Our results implicate OMA1 as a potential modulating factor in actin cytoskeleton reorganization and increased the formation of filopodia, which underscores the link between OMA1 function and suppression of metastasis. Intriguingly, this effect is specific to metastatic 21MT-1 cells, but not the non-cancerous MCF10A cells. The mitochondrial metallopeptidase OMA1 recently gained increased attention for its roles in the regulation of mitochondrial dynamics and metabolic remodeling^19^. This ATP-independent protease exists as an inner membrane (IM)-anchored, homo-oligomeric complex that is largely dormant under normal physiological conditions. A range of mitochondrial homeostatic insults triggers OMA1 activation, which results in rapid proteolytic processing of the IM GTPase, OPA1; which in turn propels fragmentation of the mitochondrial network, thereby allowing mitochondria to adapt to changing physiological demands. A growing body of evidence indicates a reciprocal link between mitochondrial morphology and cellular metabolic activity^19^.

Augmented cell motility is a fundamental aspect of increased metastasis in cancer^43,44^. Coordination between the actin cytoskeleton and microtubules are crucial for cell polarization, shape changes, and migration^43,44^. The actin cytoskeleton is believed to be the key driving force behind cell migration. At the leading edge of the cell, actin is organized in a dense meshwork, which regulates filopodia formation and promotes forward movement. In most cell types, migration is altered by interactions between the microtubule and actin cytoskeleton and it has been shown that filopodia formation at the leading edge of migrating cells is crucial for chemoattractant-induced breast cancer cell migration and invasion^39,42^. Our results suggest that OMA1 may be an important limiting factor in early molecular events that regulate breast cancer cell migration and invasion; effects likely linked to the inability of OMA1-deficient cells to initiate remodeling of the mitochondrial network due to impaired proteolysis of OPA1^45,46^. In line with this idea, several studies reported links between the mitochondrial dynamics-controlling GTPases and cellular signaling cascades of Ras^47^ and Notch^48^. Future studies are warranted to elucidate this intriguing link.

We found that under our experimental conditions, OMA1 knockdown significantly augmented proliferation of the 21MT-1 cells, as judged by elevated Ki67 expression – a change not observed in non-tumorigenic OMA1-depleted MCF10A cells. These data indicate the inverse correlation between OMA1 protein levels and cell proliferation, and increased invasive behavior of breast cancer cells, suggesting that OMA1 may potentially suppress the transition of cancer cells to metastates. It is noteworthy that our findings are in line with a recently reported p53-dependent upregulation of OMA1 expression levels in gynecological cancers – a condition that augments cisplatin-induced death of these cells^27^. An important finding of the present study is the impact of OMA1 on EMT in breast cancers. Loss of OMA1 expression significantly promoted the EMT of breast cancers with a decrease in the epithelial markers and an increase in the mesenchyme markers (Fig. 1). Importantly, EMT markers are strongly correlated to the progression of various tumors including breast to metastasis stage^49,50^. An increase in the expression of TWIST1, ZEB1, and E-cadherin has been correlated with nearly every clinically significant tumor type and has been shown to augment metastases^34,36,50,51^. One key point is that our observations in the breast cancer cells may seem discordant from the MEF experiments. This is probably because MEF cells are lacking the Tuberous Sclerosis Complex 2 (TSC2) gene, which alters PI3K-Akt signaling input to mTORC1, thereby making it constitutively active^52,53^. Such hyperactive growth factor signaling may blunt related signaling outputs and render MEF cells less sensitive to the effects of OMA1 depletion. Our results with highly confluent MEF cell cultures in which mTORC1 signaling is attenuated are consistent with this idea^54^.

Depletion of OMA1 has been shown to be at least temporarily beneficial in post-mitotic cells such as neurons or cardiomyocytes with initially compromised mitochondrial function^20,55^. However, our results suggest that the loss of OMA1, and thus the ability to remodel mitochondrial shape and metabolic outputs, can be maladaptive in rapidly dividing cells and promote neoplastic or enhance the metastatic potential of epithelial tumors. One candidate mechanism behind this effect is an elevated mitochondrial unfolded protein response. UPR^mt^ has been shown to promote glycolysis and maintain “stemness” of hematopoetic stem cells via the histone deacetylase, SIRT7, and the transcriptional activator, NRF1^56^. Elevated UPR^mt^ may therefore reflect an attempt to metabolically compensate for an inability to remodel mitochondrial morphology due to OMA1 depletion. Based on our previous^33^ and present data from experiments with *oma1*^−/−^ MEFs, it is plausible that the observed UPR^mt^-promoted metabolic effects are among the major contributors to EMT and subsequent enhancement of metastatic properties in OM.21 cells. Consistent with this postulate, recent studies have reported that stem cells^57,58^ as well as many epithelial tumors, including breast cancer^7,10,59^ display a glycolytic and/or glutaminolytic preference. These metabolic outputs are also linked to other key biosynthetic pathways such as one-carbon metabolism, whose modulation may also alter proliferative properties of a cell^60^. Additionally the idea of OMA1 levels as one of the significant predictors to clinical progression of breast cancer is supported by the *in silico* analysis of TCGA breast cancer tumors (cBioPortal database) that revealed an unfavorable risk factor for survival of patients with breast cancer in low-OMA1 mRNA expressing subgroup, in contrast to the high OMA1-expressing subgroup with better survival (Fig. 1).

In conclusion, we report that down regulation of OMA1 expression in metastatic breast cancer cells and the subsequent activation of UPR^mt^ could form the basis for promoting malignancy and metastatic progression of breast adenocarcinoma. These alterations are likely associated with metabolic remodeling towards enhanced aerobic glycolysis and glutaminolysis and are expected to correlate with poor prognosis. We anticipate that the reduced OMA1 expression-associated consequences need not be restricted to breast tumors and may possibly be recapitulated in other cancer settings as well. Testing OMA1 protein levels in cancer patients may therefore serve as a robust predictive marker for treatment responses and prognosis in development of personalized treatment strategies. Elucidation of the exact role of OMA1 in regulating tumor biology and steps of EMT will help in the development of improved anti-metastatic therapies that are useful against circulating metastatic breast cancer cells and drug resistant cancer cells.

## Materials and Methods

### Generation of stable OMA1 knockdown cell lines

21MT-1 and 21PT cell lines were a kind gift from Dr. Vimla Band at the University of Nebraska Medical Center. This cell line was isolated from metastatic pleural effusion mammary tumor and atypical ductal hyperplasia specimens respectively^61^. The 21MT-1 and 21PT cells were cultured in α-MEM media supplemented with 5% fetal bovine serum (FBS), 1% penicillin-streptomycin (PS), 1% L-glutamine, 20 mM HEPES, non-essential amino acids, sodium pyruvate (all stated reagents were from Thermo Fischer Scientific), 12.5 ng/ml epidermal growth factor (EGF) and 1 µg/ml hydrocortisone (both from Sigma-Aldrich). MCF10A (ATCC CRL-10317), human non-cancerous breast tissue cells were cultured in DMEM/F12 (Mediatech) and supplemented with 1% L-glutamine, 1% penicillin-streptomycin, 5% horse serum, 0.1 ng/ml cholera toxin, 0.5 µg/ml hydrocortisone, 10 µg/ml insulin, and 0.02 ng/µl rhEGF (all from Sigma-Aldrich). All cells were kept in aseptic conditions and grown in an incubator at 37 °C and 5% CO_2_. Cells of interest were transfected with a set of OMA1 shRNA-expressing plasmids (Origene) using Lipofectamine 3000 reagent (Thermo Fisher Scientific). The cells were allowed to recover for 24 hours in fully supplemented DMEM/F12 medium (10% FBS, 4mM L-glutamine, 4 mM glucose) without antibiotics. The medium was then replaced with fully supplemented DMEM/F12 containing 3 μg/ml puromycin. Cells were selected in this medium until a week after all the cells in the control wells (mock-transfected) were dead. Medium was replaced every 3 days. Colonies of puromycin-resistant cells were then seeded in 25 cm^2^ cell culture flasks, and tested for decrease of OMA1 expression by immunoblotting.

### Cell viability assays

The wild type and *oma1*^−/−^ mouse embryonic fibroblasts (24, kind gift from Dr. Carlos Lopez-Otin, University of Oviedo) were seeded into 12-well plates at the density of 4 × 10^4^ cells per well. DMEM medium contained 10% FCS (Thermo Fisher Scientific) and L-glutamine (4 mM), as well as glucose (10 mM), galactose (10 mM), with or without 10 µM bis-2-(5- phenylacetamido-1,2,4-thiadiazol-2-yl)ethyl sulfide (BPTES), depending on experimental condition. Every 24 hours cells from one well per condition were trypsinized with 0.05% trypsin (Life Technologies), washed with Ca-, Mg- PBS, resuspended and counted in Countess Automated Cell Counter (Life Technologies). The experiment was done in 4 biological replicates, with each replicate consisting of two technical replicates. Glucose, galactose, and BPTES were obtained from Sigma-Aldrich.

### Imaging techniques

#### Phase images

Phase images were obtained for morphology of live cells assessment using an Axiovert 40 CFL (Zeiss) and Progres C3 (Jenoptik) camera.

#### Fluorescent imaging and actin staining

At the end of the treatment, cells were fixed with 4% paraformaldehyde in PBS at room temperature for 20 min. Samples were permeabilized in 2% Triton X-100 for 15 min at room temperature. Actin 488 Ready Probe (Life Technologies) was applied according to manufacturer instructions and incubated on fixed cells at room temperature for 30 min. Nuclei were visualized with DAPI stain for 5 min, or with Hoechst 33342 (Life Technologies) stain for 10 min, incubation at room temperature in the dark in a 1 µg/ml solution. Images were obtained using Axiovert 40 CFL (Zeiss) and a Progres C3 (Jenoptik) camera with an X-Cite series 120Q (Lumen Dynamics) lamp utilizing FITC or DAPI filter (Chroma).

#### Membrane potential measurement

Cells were seeded overnight in 6-well plates at 500,000 cells per well. Prior to the experiment, cells were either treated with vehicle (DMSO) or CCCP (final concentration 2uM) for 1 hr. Following the treatments, cells were trypsinized with 0.05% trypsin, washed 3x with PBS and resuspended in 1 ml PBS for staining. Cells were stained on ice in 50 nM TMRM (Sigma) for 15 min, and subsequently assayed on Cytek DxP10 flow cytometer (Cytek Biosciences). TMRM fluorescence was measured using 561 nm excitation, and 580/20 nm emission; 50,000 cells were assayed in each experiment. Results were analyzed and plotted using FlowJo 10 software (FlowJo).

#### Mitochondrial imaging

Mitochondrial morphology was assessed using MitoTrackerR Red CMXRos (Life Technologies) staining according to manufacturer’s instructions. Briefly, 24 hr following the seeding of cells, the old culture media was removed and replaced with serum-free, phenol red-free DMEM containing 300 nM MitoTrackerR probe and incubated at 37 °C for 30 min. Cells were then washed three times with warm 1X PBS and serum free, phenol-red free DMEM was added for viewing with an Olympus FV500 Inverted Confocal Microscope.

#### Quantitative real-time PCR

qRT-PCR was performed using standard qRT-PCR program on ABI 7900HT qRT-PCR cycler (Applied Biosystems), with validated qRT-PCR primers (Realtimeprimers.com). Expression data for *AFG3L2*, *CLPP*, *SPG7* and *HSPD1* were normalized using qRT-PCR for Actin B. SYBR-Green qPCR mix was obtained from Life Technologies. The experiment was done in 3 biological replicates, each consisting of 4 technical replicates.

#### Western blotting

Whole cell lysates from cell cultures was prepared using RIPA buffer (1X PBS containing 1% IGEPAL CA-630, 0.5% sodium deoxycholate, 0.1% sodium dodecyl sulfate (SDS), 1 mM phenylmethylsulfonyl fluoride (PMSF) and protease inhibitor cocktail). Cells were washed three times with ice cold 1X PBS, lysed in RIPA buffer on ice for 10 min. Samples were then centrifuged at 10,000 × g for 20 min at 4 °C, supernatant was transferred to 0.2 μM microcentrifuge filters (ThermoFisher Scientific), and centrifuged at 10,000 × g for 10 min at 4 °C to collect clear cell lysate. Protein concentrations were determined using Coomassie Plus Assay reagent (Pierce). 10 μg of total protein was separated by 7.5% SDS-polyacrylamide gel electrophoresis and transferred to Immobilon P membranes (Millipore) using transfer buffer (25 mM Tris, 192 mM glycine, 10% methanol). Membranes were blocked with 5% skimmed milk for 2 h at room temperature (RT) followed by incubations with either anti-EMT markers IgG panel (GeneTex GTX300096, 1:1000), or anti-OMA1 IgG (Aviva Systems Biology ARP52818_P050, 1:1000), or anti-AFG3L2 IgG (Aviva Systems Biology ARP46780_P050, 1:1000), or anti-HSPD1 (Aviva Systems Biology AVARP09014_P050, 1:1000), or anti-HIF1α IgG (Aviva Systems Biology ARP38054_P050, 1:1000) or anti-GAPDH IgG (Millipore ABS16, 1:4000) for overnight at 4 °C. Membranes were then incubated with HRP-linked goat anti-rabbit IgG (Santa Cruz Biotechnology sc2054, 1:4000) for 1 h at RT. The antigen-antibody complex was detected using ECL and the images were captured on autoradiography films (ECL Hyperfilm, Pierce). The immunoreactive signals were quantified by densitometry using Image Studio™ Lite Software v.4.0 (LI-COR Biosciences).

#### *In vitro* migration assay

21MT-1, 21PT, or MCF10A cells (control and shOMA1) were plated into 12 well plates as confluent monolayers. Three vertical scratches were made per well (using a 10 µl pipette tip), and cell debris was washed away with 1X PBS prior to capturing the zero time photos. Additional photos were taken at 24 and 48 hours time points. The cells were supplemented with complete 21MT-1 media for the entire migration assay. ImageJ software was used to quantify the scratch width (arbitrary units) changes at each time point. A total of 27 scratch width data points were taken for each sample type at each time point (3 scratches per well x3 pictures per scratch x3 biological duplicates per sample type).

#### Bioenergetic profiling

Metabolic quantification of cells was measured using a Seahorse XFe24 Extracellular Flux Analyzer (Seahorse Bioscience) in XFe24 microplates. Cells were seeded at the density of 5 × 10^4^ cells per well 24 hours before the experiment in Seahorse medium (Agilent Technologies/Seahorse Bioscience) supplemented with L-glutamine (4 mM), as well as glucose (10 mM), galactose (10 mM), BPTES (10 µM) and pyruvate (2 mM), depending on experimental condition. After 24 hours, the cells were washed twice and supplemented with 0.5 ml fresh Seahorse medium containing L-glutamine (4 mM), glucose (10 mM), galactose (10 mM), BPTES (10 µM), and pyruvate (2 mM), depending on experimental condition, and kept for 1 hour in non-CO_2_ incubator at 37 °C. The OCR was measured under basal conditions and addition of olygomycin (1 µM), FCCP (1 µM), and rotenone/antimycin A (0.5 µM). Measurements were performed using standard Mito Stress protocol on XF^e^24 Extracellular Flow Analyzer (Agilent Technologies). Oligomycin, FCCP, and rotenone/antimycin A were obtained as a part of Mito Stress Kit (Agilent Technologies/Seahorse Bioscience). Glucose, galactose, BPTES, L-glutamine, and pyruvate were obtained from Sigma-Aldrich. Extracellular acidification rates (ECAR) were measured using the Seahorse XF Cell Glyco Stress Test using successive injections of glucose (10 mM), oligomycin (1 µM), and 2-DG (50 mM). Three measurements were taken before and after each injection and mixing cycle. Rates were normalized to non-glycolytic acidification rate by well. The experiment was done in 4 biological replicates, with each replicate consisting of 3–4 technical repeats per condition.

#### Bioinformatics and statistical analyses

Expression of OMA1 mRNA, evaluated from RNA-Seq data, available for breast cancer tumors, was downloaded from TCGA provisional dataset and UCSC Xena websites. OMA1 mRNA expressed as OMA1 mRNA expression, Batch Normalized/Merged from Illumina HiSeq_RNA SeqV2 syn4976739, generated by TCGA, was plotted using the Box and Whisker plot^62^. The Box and Whisker plot depicts normal distribution of OMA1 mRNA and determines the median and quartiles in a statistical population.

Utilizing the TCGA data, Kaplan-Meier survival curves were generated by dividing tumors into non-overlapping upper and lower groups based on OMA1 mRNA values up to 25^th^ percentile as low and above 25^th^ as high^31,32^. Log-rank (Mantel-Cox) statistical test was used to compare the survival distribution of these two samples.

Statistical analyses were performed using Microsoft Excel 2013 Analysis ToolPak and GraphPad Prism 4 software. In each case, the results of at least three independent experiments were analyzed using one-way ANOVA or Student’s *t*-test. The *p* values <0.05 were considered statistically significant.

### One-sentence summary

Breast cancer patients with low mitochondrial protease OMA1 expression have a shorter survival time; depletion of OMA1 enhances formation of filopodia, increases cell proliferation, and induces epithelial-mesenchymal transition, thereby stimulating migratory properties of metastatic breast cancer cells.

## Supplementary information

Supplementary Information
