# Supplementary material for: Depletion of mitochondrial protease OMA1 alters proliferative properties and promotes metastatic growth of breast cancer cells
Source: Sci Rep. 2019 Oct 14;9:14746. doi: 10.1038/s41598-019-49327-2 (PMC6791882; doi:10.1038/s41598-019-49327-2)
Supplement: Supplementary file 1 — Supplementary Information [file 41598_2019_49327_MOESM1_ESM.pdf]

Depletion of mitochondrial protease OMA1 alters proliferative properties and promotes metastatic growth of breast cancer cells

Amita Daverey[1§], Roman M. Levytskyy[2§], Samantha Swenson[2], Stephen L. Hayward[1], Kimberly M. Stanke[1], Martonio Ponte Viana[2], Madhusudhanan Narasimhan[3], Oleh Khalimonchuk[2, 4,5,6]*, Srivatsan Kidambi[1, 5,6,7,8,9]*

1: Department of Chemical and Biomolecular Engineering, University of Nebraska, Lincoln, NE, United States

2: Department of Biochemistry, University of Nebraska, Lincoln, NE, United States

3: Department of Pharmacology and Neuroscience, Texas Tech University Health Sciences Center, Lubbock, TX, United States

4: Nebraska Redox Biology Center, University of Nebraska, Lincoln, NE, United States

5: Fred & Pamela Buffett Cancer Center, University of Nebraska Medical Center, Omaha, NE, United States

6: Nebraska Center for Integrated Biomolecular Communication, University of Nebraska, Lincoln, NE, United States

7: Nebraska Center for the Prevention of Obesity Diseases, University of Nebraska, Lincoln, NE, United States

8: Nebraska Center for Materials and Nanoscience, University of Nebraska, Lincoln, NE, United States

9: Mary and Dick Holland Regenerative Medicine Program, University of Nebraska Medical Center, Omaha, NE, United States

[§] A.D. and R.M.L. contributed equally to this work

Running title: OMA1 in breast cancer metastasis

Key words: Breast cancer, Mitochondria, OMA1, EMT markers, cell migration

*Address correspondence to:

Srivatsan Kidambi, skidambi2@unl.edu

Oleh Khalimonchuk, okhalimonchuk2@unl.edu

Supplementary Figure Legends

Figure S1: Screening of siRNA for OMA1 knockdown. The endogenous levels of OMA1 protein. Whole cell lysates prepared from MCF10A and 21MT1 cells transfected with shRNAs targeted against OMA1 (OMA1Sh2, OMA1Sh3) or scrambled shRNA (scrambled) were subjected to immunoblotting for OMA1 and GAPDH (loading control).

Figure S2. OMA1 depletion in metastatic breast cancer cells promotes filopodia formation. Live phase contrast and fluorescent images of control and OMA1 knockdown (KD) 21MT-1 (OM.21) cells. Unlike the 21MT-1 cells, OM.21 cells exhibited characteristic filopodia-like structures after overgrowth. 21MT-1 and OM.21 cells were stained for Actin with Alexa Fluor 488-conjugated phalloidin and Nuclei with Hoeschst (blue) imaged by confocal microscopy actin. Scale bar, 20 μm.

Figure S3. OMA1 deficiency enhances invasiveness of breast cancer cells by inducing epithelial to mesenchymal transition (EMT) (A) Representative western blot of extracts from atypical ductal hyperplasia (21PT) cells after transfection with an shRNA scrambled control, and shRNA directed against OMA1. Steady-state levels of the protease were detected by immunoblotting with anti-OMA1 antibody. The GAPDH served as a loading control and was visualized with the respective antibody. **(B)** Proliferation of control 21PT and OP.21 cells seeded after overgrowth of 7 days at 100% confluence. Data represent the mean ± S.D. of n=3 biological replicates; **p*<0.05, ****p*<0.001. **(C)** Live phase contrast images of 21PT cells before and after transfection with OMA1-targeting shRNA at 24h and 48h after seeding in normal culture conditions. Unlike the control 21PT cells, OP.21 cells readily exhibit characteristic filopodia-like structures (marked by black arrows). Scale bar, 20 μm. **(D)** The prevalence of cells with filopodia-like protrusions was semi-quantitatively analyzed in 21PT and OP.21 cells two days after re-seeding on 6-well plates. Each well was randomly imaged in 3-4 fields of view, each containing 40-75 cells. Of the cells in the field of view the number of cells with long filopodia and cells with filopodia were counted. The data was plotted as a scatter plot wherein each point represents percentage of cells with filopodia of total cells in one field of view. n=3; ****p*<0.001. **(E)** Wound closure assay data detailing the migration of indicated cells out from a confluent monolayer onto a featureless scratch. Representative phase images of control and OP.21 cells are shown. Scale bar, 20 μm. **(F)** Quantitative analysis of migratory properties of control and OP.21 cells. The extent of wound closure has been calculated as follows: % Wound Closure = [(Area at 0h – Area at 24 or 48h)/ Area at 0h] x 100%. Bars show mean data ± S.D. of 3 biological replicates; ****p*<0.001 by unpaired *t*-test. **(G)** qRT-PCR analysis of the indicated EMT marker genes expression in control and OP.21 cells after 2 days in culture. The expression levels were normalized to GAPDH transcript and to control cells. Data is presented as mean ± S.D. from 3 biological replicates. **p*< 0.05; ***p*< 0.01; ****p*< 0.001 by Student's *t*-test.

Figure S4: OMA1 depletion in 21MT-1 cells alters their bioenergetic and metabolic properties. (A) *Top panel* shows a representative western blot of extracts from the metastatic pleural effusion mammary tumor 21MT-1 cells after transfection with an shRNA scrambled control, and shRNA directed against OMA1 (OM.21 cells). Steady-state levels of the protease were detected by immunoblotting with anti-OMA1. The GAPDH served as a loading control and was visualized with the respective antibody. *Bottom panel* shows quantitative assessment of the above immunoblots. Error bars indicate mean data ± S.D. n=3 independent experiments; ****p*<0.001 by unpaired *t*-test. **(B)** Representative oxygen consumption rates (OCR) in control and OM.21 cells under basal, oligomycin A (OLA), FCCP, and antimycin A/rotenone (AA+Rot.) stimulated conditions. Cells were cultured in the medium containing 10mM galactose. **(C)** Respiratory control ratios in control and OM.21 cells cultured in 10 mM galactose medium. Data

represent mean values ± S.E.M. (n=3 biological replicates); *$p<0.05$, by t-test. **(D)** Growth rates of control and OM.21 cells under glutamate pathway inhibition condition. Cells were cultured in 10mM galactose-containing medium in the presence of 10 μM BPTES for the indicated periods of time and the number of viable cells at each time point has been assessed. Data represent the mean ± S.D. (n=3 biological replicates); *$p<0.05$, ***$p<0.001$, by unpaired t-test. **(E)** The prevalence of cells with protrusions was analyzed in 21MT-1 and OM.21 cells two days after re-seeding on 6-well plates. Each well was randomly imaged in 3-4 fields of view, each containing 40-75 cells. Of the cells in the field of view the number of cells with filopodia-like protrusions were counted. The data was plotted as a scatter plot where each point represents the average filopodia size of total cells in one field of view.

Figure S5. Mitochondrial membrane potential is decreased in OMA1-deleted MEF cells. Representative flow cytometry analysis histograms of control and *oma1*$^{-/-}$ MEF cells incubated with 50 nM TMRM under basal, and CCCP-induced uncoupling conditions. 50,000 cells were assayed for TMRM fluorescence in each experiment.

Figure S6: Source data for Figure S1.

Figure S7: Source data for Figure 2A and Figure S4.

Figure S8: Source data for Figure 6B.

Figure S9: Source data for Figure 7B.

Figure S10: Source data for Supplementary Figure S3A.

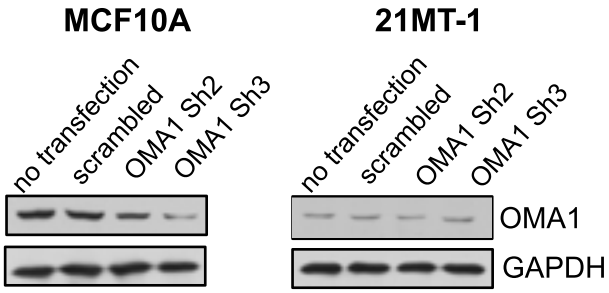

Figure S1: Screening of siRNA for OMA1 expression.

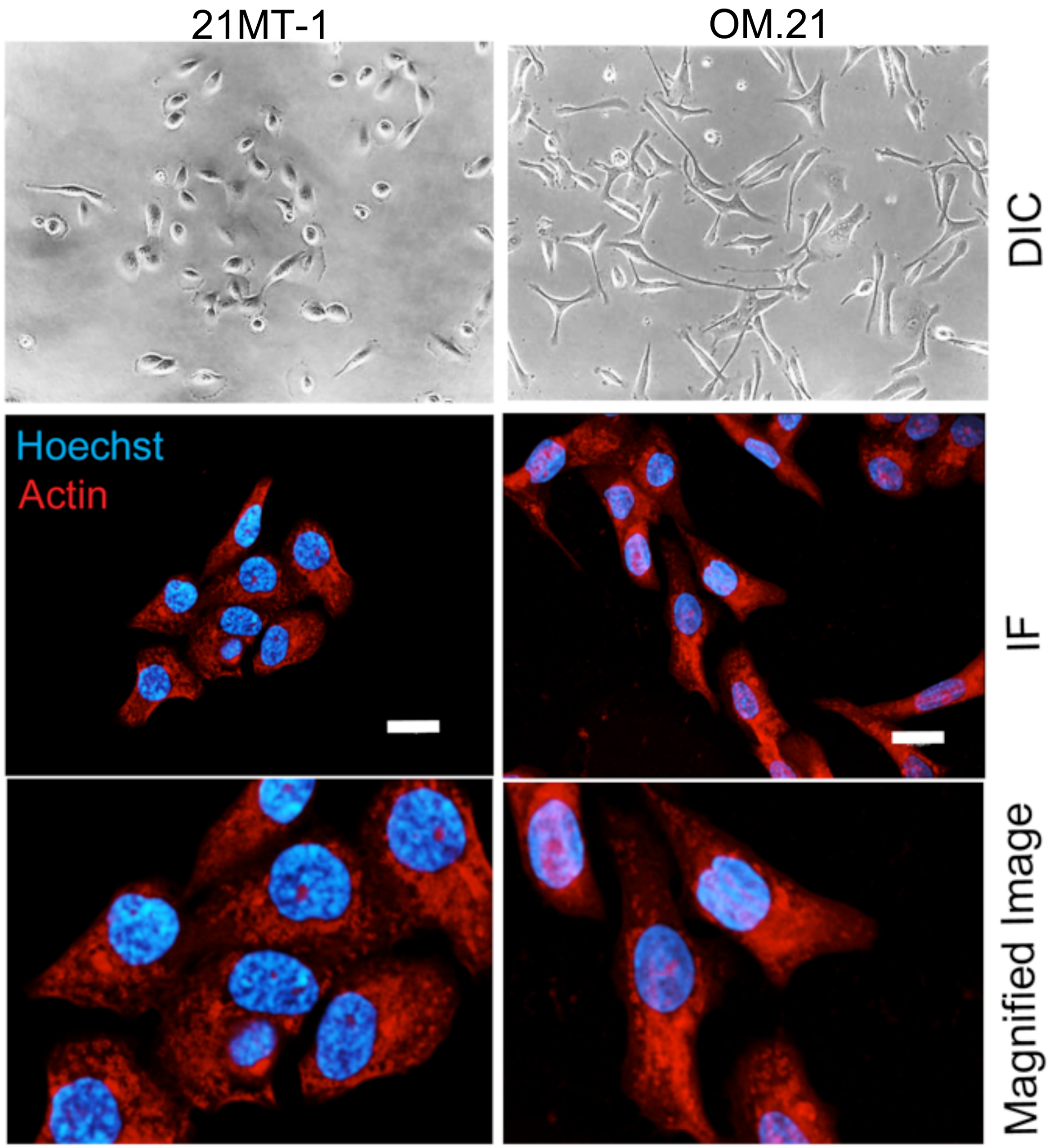

Figure S2. OMA1 depletion in metastatic breast cancer cells promotes filopodia formation

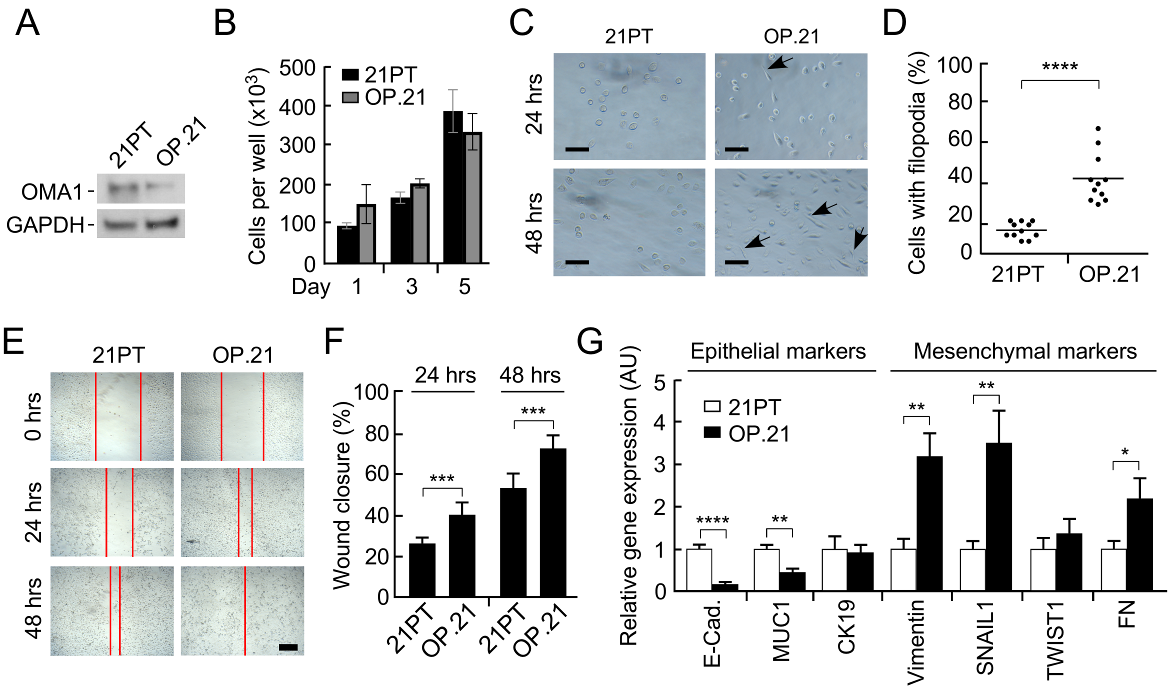

Figure S3. OMA1 deficiency enhances invasiveness of breast cancer cells by inducing epithelial to mesenchymal transition (EMT)

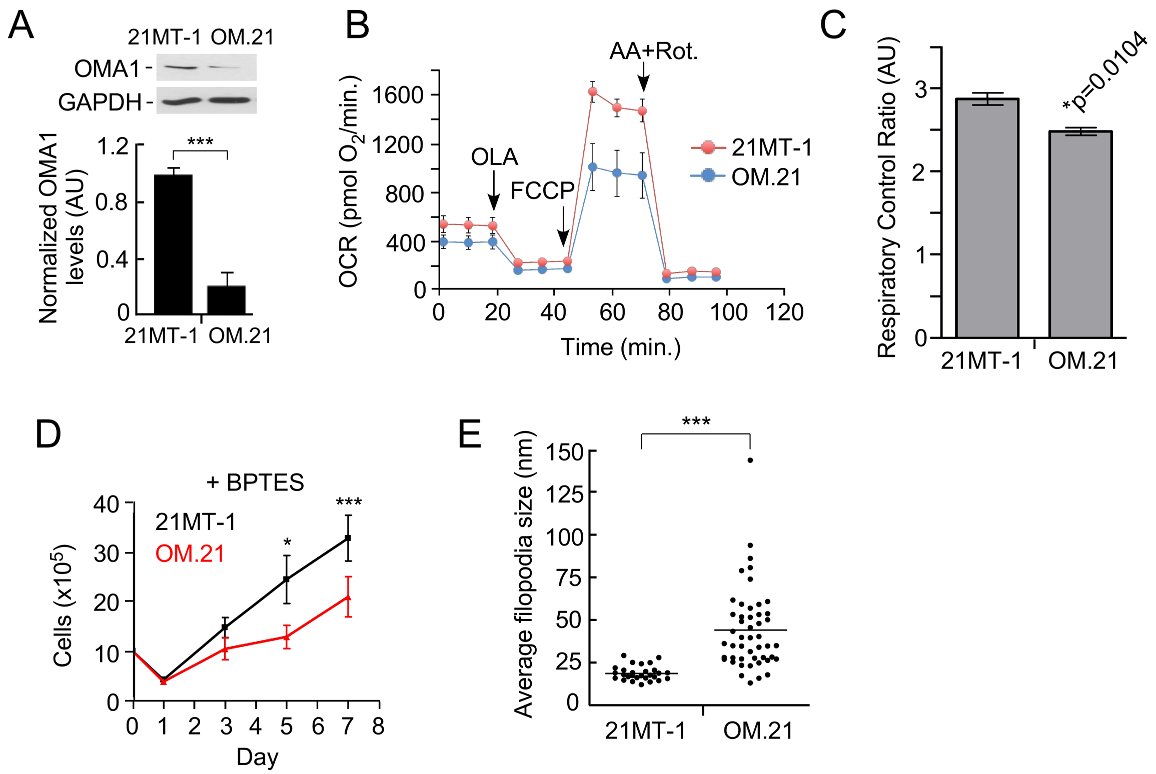

Figure S4: OMA1 depletion in 21MT-1 cells alters their bioenergetic and metabolic properties.

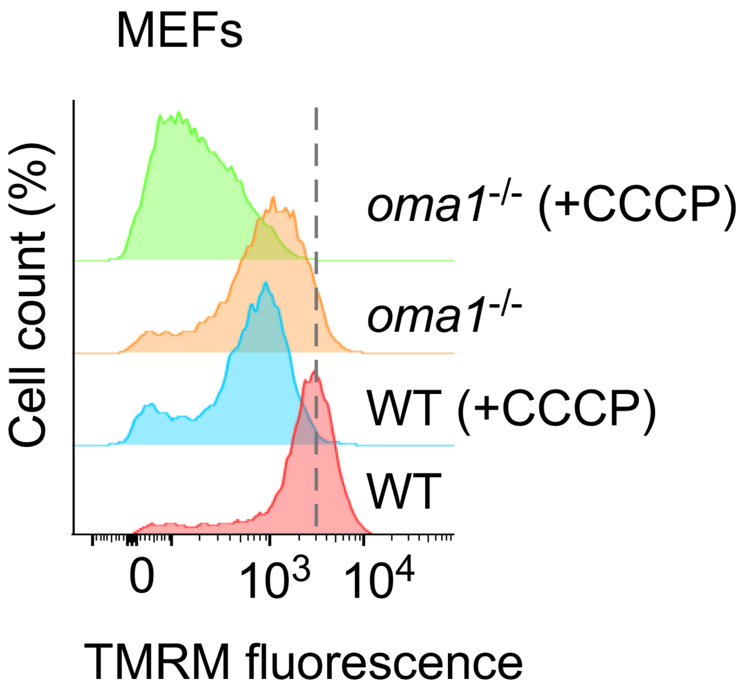

Figure S5. Mitochondrial membrane potential is decreased in OMA1-deleted MEF cells.

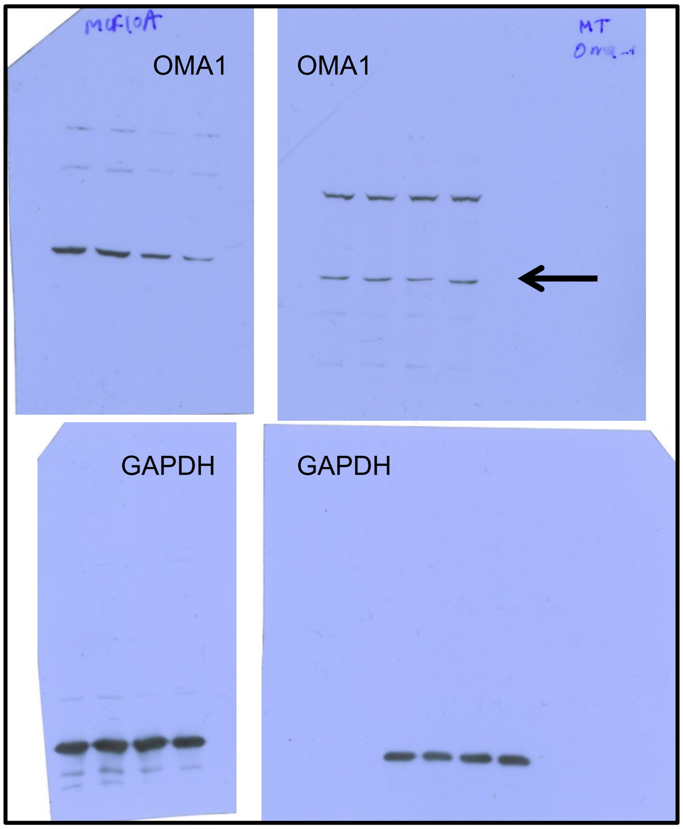

Figure S6: Source data for Figure S1.

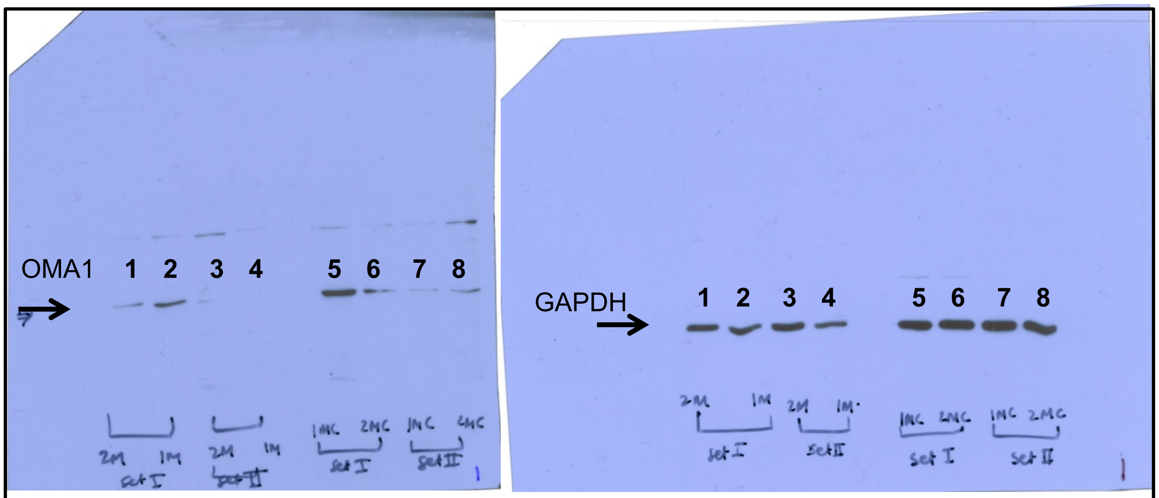

Lane 1: OM.21 Set I
Lane 2: 21MT-1 Set I
Lane 3: OM.21 Set II
Lane 4: 21MT-1 Set II
Lane 5: MCF10A Set I
Lane 6: OM.10A Set I
Lane 7: MCF10A Set II
Lane 8: OM.10A Set II

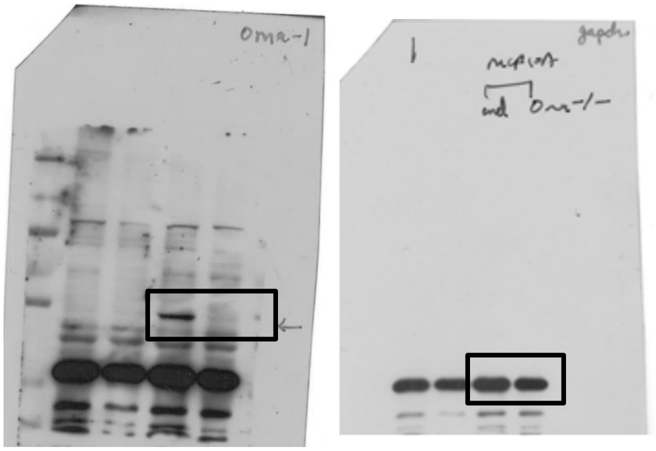

Figure S7: Source data for Figure 3A and Figure S4.

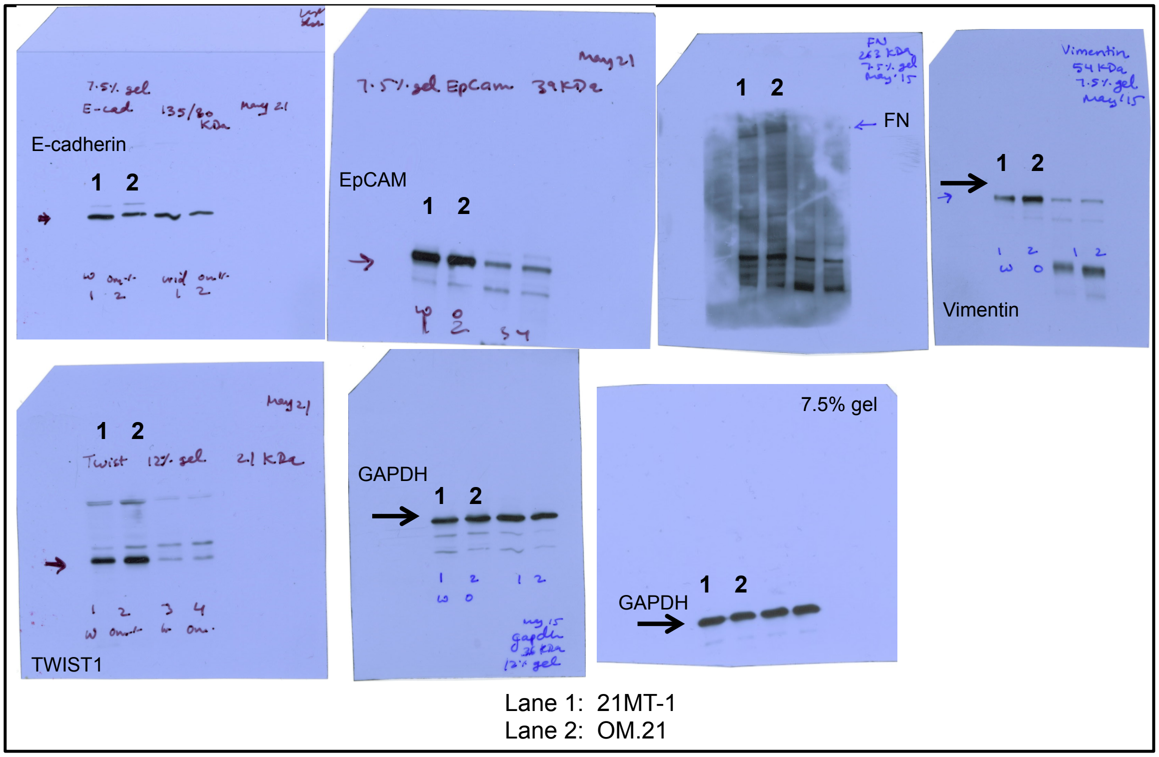

Figure S8: Source data for Figure 6B.

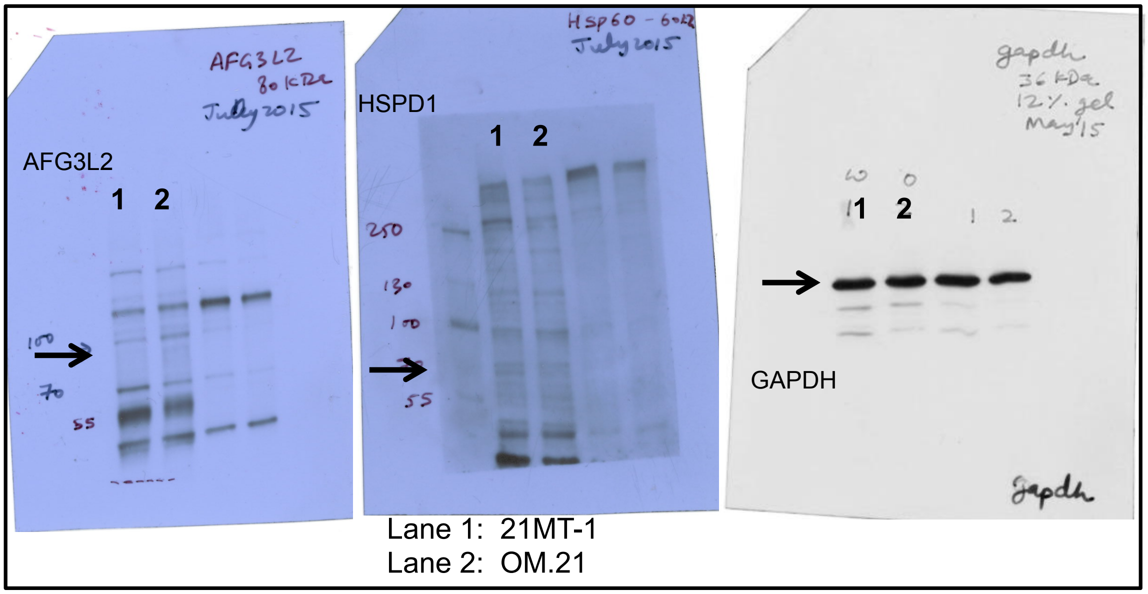

Figure S9: Source data for Figure 7B.

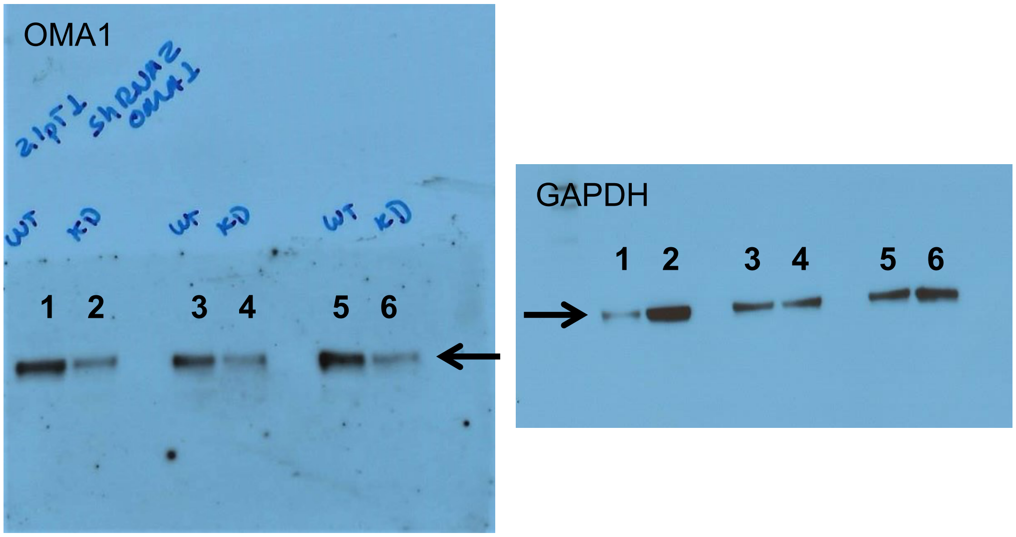

Lane 1: 21PT Set I
Lane 2: OP.21 Set I
Lane 3: 21PT Set II
Lane 4: OP.21 Set II
Lane 5: 21PT Set III
Lane 6: OP.21 Set III

Figure S10: Source data for Supplementary Figure S3A.
